# Application of the SwitchSense Technique for the Study of Small Molecules’ (Ethidium Bromide and Selected Sulfonamide Derivatives) Affinity to DNA in Real Time

**DOI:** 10.1021/acs.jpcb.2c03138

**Published:** 2022-09-15

**Authors:** Sandra Ramotowska, Paulina Spisz, Jakub Brzeski, Aleksandra Ciesielska, Mariusz Makowski

**Affiliations:** † Faculty of Chemistry, University of Gdańsk, Wita Stwosza 63, Gdańsk 80-308, Poland; ‡ Department of Chemistry, University of Pittsburgh, Pittsburgh, Pennsylvania 15260, United States

## Abstract

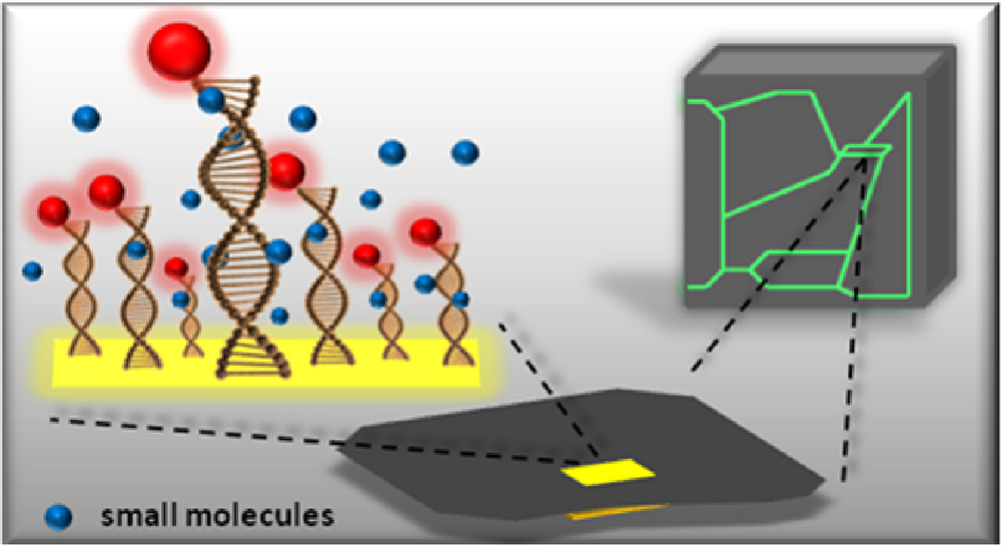

The discovery and introduction of the switchSense technique in the chemical laboratory have drawn well-deserved interest owing to its wide range of applications. Namely, it can be used to determine the diameter of proteins, alterations in their tertiary structures (folding), and many other conformational changes that are important from a biological point of view. The essence of this technique is based on its ability to study of the interactions between an analyte and a ligand in real time (in a buffer flow). Its simplicity, on the other hand, is based on the use of a signaling system that provides information about the ongoing interactions based on the changes in the fluorescence intensity. This technique can be extremely advantageous in the study of new pharmaceuticals. The design of compounds with biological activity, as well as the determination of their molecular targets and modes of interactions, is crucial in the search for new drugs and the fight against drug resistance. This article presents another possible application of the switchSense technique for the study of the binding kinetics of small model molecules such as ethidium bromide (EB) and selected sulfonamide derivatives with DNA in the static and dynamic modes at three different temperatures (15, 25, and 37 °C) each. The experimental results remain in very good agreement with the molecular dynamics docking ones. These physicochemical insights and applications obtained from the switchSense technique allow for the design of an effective strategy for molecular interaction assessments of small but pharmaceutically important molecules with DNA.

## Introduction

**Figure 1:**
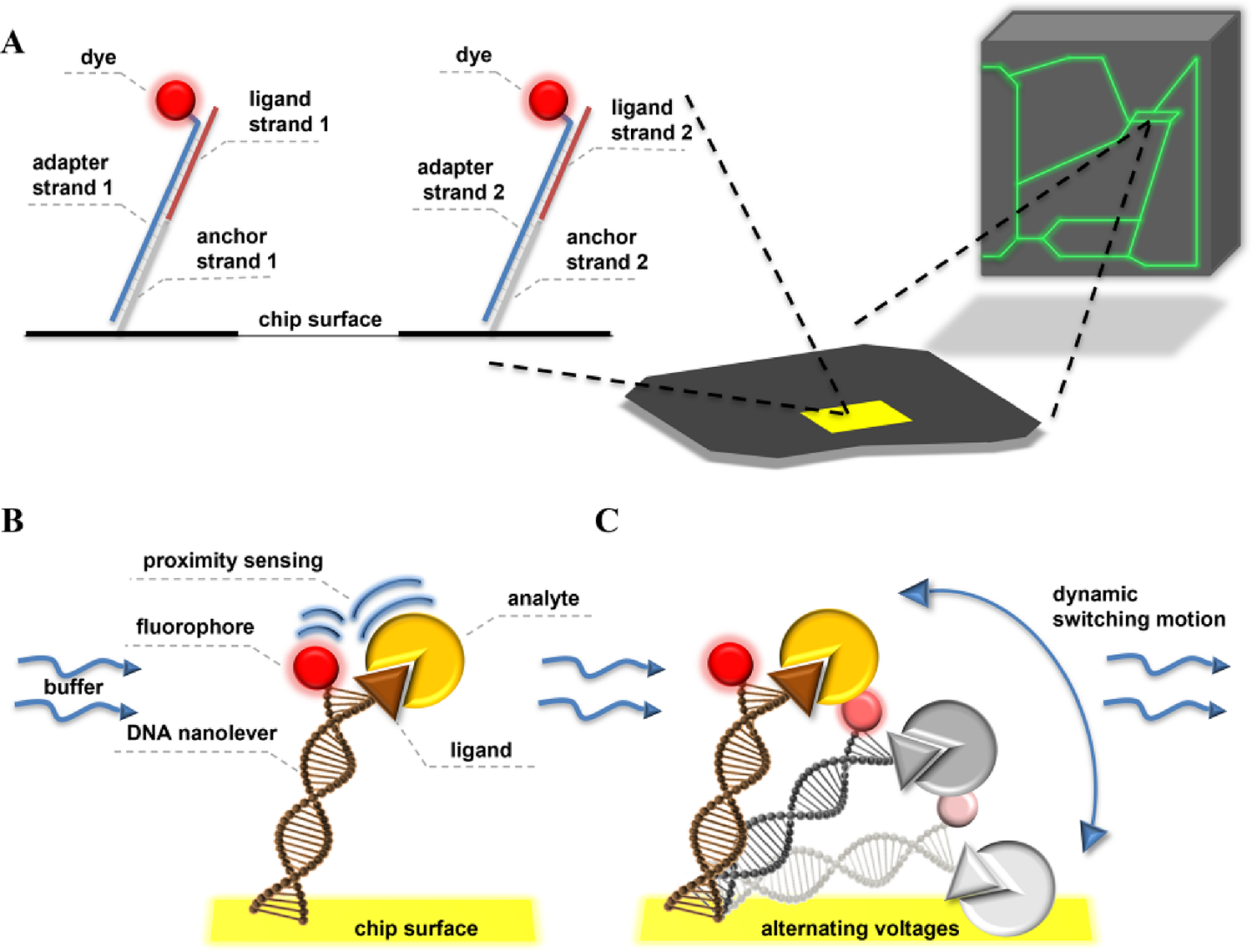
Principle of the heliX (Dynamic Biosensors) apparatus. (A) Scheme of the modified chip surface. Schematic presentation of the measurement system in (B) static mode and (C) dynamic mode.

Why is the search for new, effective methods of studying the interactions of compounds with potential pharmacological applications with DNA so crucial? Each cell in the human body contains one molecule of genomic DNA and various proteins in numerous copies. Through the genetic information collected in the DNA, a damaged protein can be biosynthesized. On the other hand, DNA damage beyond repair capacity leads to cell death. This is why DNA is a target of many therapies and why it is so important to study the interaction of potential pharmaceuticals with this biomolecule. Scientists from all over the world seek new tools and applications of accessible methods for the precise description of physicochemical and biological phenomena. One of these tools may be the recently developed technique called switchSense.^1−5^ This technology uses chips with an electrically switchable gold surface covered with DNA nanolevers, which enables the characterization of intermolecular interactions in real time (Figure 1). The single nanolever consists of an anchor strand that is covalently attached to a chip surface and an adapter strand terminated with a fluorescence dye. The sequence binds to the anchor strand due to complementarity. The sequences are specially selected to create a stable double strand even up to 80 °C. The last component is a ligand strand that can bind different types of ligands. To extend the chips’ lifetime and increase the possibility of their functionalization, they have been adopted to be regenerable and thus reusable. This also allows for the reduction of the cost of the measurements, which is of utmost importance while working with biologically active compounds.

The switchSense combines sensitive kinetic research with structural information, such as the shape, size, and conformation of biomolecules, enabling the understanding of interactions at a molecular level. Dedicated electronics manage the electric actuation of fluorescently labeled DNA nanolevers placed on the biochip surface using the phenomenon of electrically triggered time-correlated single-photon counting (E-TCSPC). The nanolevers are introduced into a controlled movement by changing the voltage on the surface of the gold electrodes. When the interaction occurs, nanoprobe oscillations and/or dye fluorescence change, which are then used to determine the number of kinetic and biophysical parameters. To obtain data on molecular interactions, the apparatus combines automatic fluid distribution, measurement mode control (static or dynamic), temperature control, chip management, and the introduction of numerous modifications to the nanolever system. Moreover, using the microfluidics system, low sample consumption and biochip regeneration make the switchSense technique economical and environmentally friendly, which is in agreement with ″The 12 Principles of Green Chemistry″.^6^

SwitchSense has been successfully used to determine numerous physicochemical properties and quantities such as diameters of proteins, protein folding, and conformational changes.^7^ Its applicability can also be extended to study the enzyme activity and the influence of ions on nucleic acid folding, analyze the monomeric and trimeric states of TNF-α (tumor necrosis factor-alpha), and detect carcinogenic water pollutants.^8−11^ Furthermore, it has proven useful and effective in obtaining the binding and dissociation kinetic parameters of molecules such as proteins or polyamides to nucleic acids^8,12−15^ and interactions of small molecules with the human serum albumin*.*^16^

Based on the fact that DNA acts as a molecular target for many of the pharmaceuticals used in a variety of therapies, e.g., anticancer treatment, extending the application of switchSense seems to be desirable. This technique allows the assessment of both association and dissociation processes for ligand–analyte interactions. The determination of the binding strength is one of the factors that provide a solid justification for further research, including biological study. Our group has recently started working on adapting this technique for the study of the mechanism and strength of binding of small molecules directly to the DNA chain. The preliminary studies were performed with a well-known DNA intercalator: ethidium bromide (EB, a model molecule). The EB is described by a high value of the binding constant to the DNA, and its thermodynamic characteristic of binding to the said biomolecule has been well described in other types of experiments.^17−21^ The sulfonamides have been selected as the main research object due to their proven antibiotic and anticancer properties.^22−27^ Therefore, the second small compound that has been selected for the research was sulfathiazole (STZ), a chemotherapeutic agent with a strong bacteriostatic effect. Its interaction with DNA in solutions was proven by our group using spectrophotometric titration.^28^ It was previously confirmed that STZ was also a promising ligand for the formation of complexes with transition metal ions (e.g., Ru(III)) and that the complexation improved its antimicrobial and anticancer properties.^28^ As for further research objects, two sulfonamide derivatives differing in the alkylamino substituent length, 4-amino-*N*-(2-aminoethyl)benzenesulfonamide (NethylS) and 4-amino-*N*-(3-aminopropyl)benzenesulfonamide (NpropylS), were selected. The physicochemical and complex forming properties of said compounds were recently determined by our group.^29^

Understanding the mechanism of DNA–drug interactions is crucial in the drug design process as well as in biological activity studies. A combination of both innovative experimental technique and well-known computational methods to determine the binding mode and strength of the interaction of small molecules to the DNA chain was used in the present paper. Such an approach might prove extremely important as both primary and complementary analytical tools for rather costly and time-consuming *in vivo* studies. Therefore, in this paper, we (i) present a never reported route of obtaining the optimal methodology for studying the kinetics of binding of small molecule compounds to DNA using the switchSense technique; (ii) describe the affinity of studied sulfonamides to a selected DNA double-strand sequence by determining parameters such as the association/dissociation rate and binding constant for the sulfonamide–DNA adduct; and (iii) discuss the possible mode and binding sites of studied sulfonamides to the particular base pair sequence used in this study. Our results are promising, and the technique has the potential of becoming a powerful tool for the study of the affinity of pharmaceuticals to biomolecules in real time. We do believe that this innovative use of switchSense technology would facilitate the research on biologically active compounds targeting nucleic acid.

## Experimental Section

### Samples and Measurements

The ethidium bromide and sulfathiazole were purchased from Sigma Aldrich. The NethylS and NpropylS were synthesized previously by our group. The synthesis pathway for the NethylS and NpropylS procedure was described elsewhere.^29^ All buffers and solutions (PE40 buffer ×10, regeneration, passivation ×10, EDTA, chip, and standby solutions) together with 96-well plates and 1.3 and 10.0 mL autosampler vials with caps dedicated to the heliX instrument were delivered by Dynamic Biosensors GmbH (Planegg, Germany). The buffers and solutions requiring dilution were prepared from double-distilled and additionally filtered (2 μm) water. The analyte (EB, STZ, NethylS, and NpropylS) solutions were prepared and measured in several concentrations, i.e., the ethidium bromide from 10^–6^ to 10^–9^ M (dilution factors 10 and 2), the sulfathiazole from 1 × 10^–4^ to 1.25 × 10^–5^ M (dilution factor 2), the NethylS from 2 × 10^–4^ to 2.5 × 10^–5^ M (dilution factor 2), and NpropylS from 8 × 10^–4^ to 1 × 10^–4^ M (dilution factor 2). The EB samples were prepared using the PE140 buffer (pH 7.4; 10 mM Na_2_HPO_4_/NaH_2_PO_4_, 140 mM NaCl, 0.05% Tween20, 50 μM EDTA, 50 μM EGTA) and also using the PE40 buffer (pH 7.4; 10 mM Na_2_HPO_4_/NaH_2_PO_4_, 40 mM NaCl, 0.05% Tween20, 50 μM EDTA, 50 μM EGTA), while for sulfonamides, the PE40 buffer was used. To reduce the evaporation of samples during the measurement, the plates were sealed.

### The Measuring System

The measuring system is made of a DNA probe terminated with a fluorophore (red, dye A) immobilized on the gold biosurface of the chip (standard adapter, HeliX-ADP-2-0). The DNA fragment is a 96 bp (base-pair) sequence where both the adapter (5′-48 bases + TAG TGC TGT AGG AGA ATA TAC GGG CTG CTC GTG TTG ACA AGT ACT GAT-3′) and ligand-free strands (5′-ATC AGT ACT TGT CAA CAC GAG CAG CCC GTA TAT TCT CCT ACA GCA CTA-3′) are distinguished. The first 48 bp is internal company secret information. The strands were provided by the manufacturer in a prehybridized form as chip and standby solutions. The measurement system was the same on spots 1 and 2.

### Dynamic and Static Modes of Measurements

The experiments were performed using two modes: dynamic and static. In the first of them, there was a need to use the stepwise measurement approach, while in the static mode, the experiments were performed using the methods provided by manufacturers (Standard Kinetics v46, weak binder kinetics). The first step of all experiments was functionalization. The adapter concentration was 1 × 10^–7^ M, and the time of this process was 200 s. The proper kinetics analysis was our next step. Depending on the selected mode and method, the association and dissociation times differed as follows: (i) in the dynamic mode and for the weak binder method, these were equal to 30 and 60 s, respectively, (ii) while in the static mode and for the standard method, these were equal to 60 and 300 s, respectively. The flow rate in all cases was 200 μL/s. The dissociation process was carried out until the analyte was completely washed out of the system by the buffer. The LED (light-emitting diode) power was 2. The analysis was performed for the five concentration variants, namely, 0 (blank) and the remaining four (listed above) depending on the tested compound. The blank was performed before and after a series of concentrations. The analyses were performed in either the PE140 or PE40 buffer; these buffers differ in NaCl concentration and hence ionic strength. All of these parameters were chosen based on optimization. The measurements in the various flow rates, in the range of 50 to 500 μL/s, and time variants of the dissociation and association process have been performed.

All variants of measurements have been registered at three temperatures: 15, 25, and 37 °C. To check the status and parameters of the used chips, a chip test procedure was performed before and after the measurement. The chip tests were carried out using the method provided by the manufacturers (v3) with an inflection point of 0.15 and a temperature of 25 °C.

### Result Analysis

The results were analyzed using the Helix software (v1.7.0). All curves of response as fluorescence change during the dissociation and association processes as one data set (data for five different concentrations) were fitted with the 1:1 interaction model expressed by eqs 1 and 2, respectively.
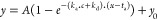
1
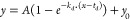
2where *A* is a signal amplitude; *t*_a_ and *t*_d_ are start times assigned to the association and dissociation processes, respectively; *y*_0_ is a baseline; *c* is a concentration; and *k*_a_ and *k*_d_ are association and dissociation rates, respectively. Considering eqs 1 and 2, the association constant (*K*_A_) could be calculated following eq 3 at the equilibrium:
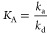
3

### Computational Methods

The equilibrium structures of all analytes (EB and sulfonamides) were obtained by geometry optimizations employing the wB97XD^30^ hybrid-functional including empirical dispersion and the 6-311++G(2d,2p) Pople-type basis set.^31^ Force constants and vibrational frequencies were then calculated to ensure that optimized structures are true minima on the potential energy surface. The aqueous environment (ε = 78.3553) of the solution was approximated by employing the CPCM^32^ solvation model in all the above-mentioned calculations. The Cartesian coordinates of the equilibrium structures of all compounds considered here are collected in Table S1. All quantum chemical calculations were carried out using the GAUSSIAN16 (Revision C.01)^33^ computational package.

Because the experimental structure of the DNA helix used in this work is not well-known and the only data on the structure is its sequence, the Nucleic Acid Builder (NAB)^34^ was used to build an initial structure for further calculations. The B-type conformation of the DNA was assumed at this step, as it is the most abundant in cells.^35^ In the next step, the OL15 force field^36^ was used to obtain the initial parameters (topology and coordinates) for MD simulations. The DNA double helix was solvated with 95,963 TIP3P model^37^ water molecules and placed in a truncated octahedral periodic box with an edge length of 157 Å and a minimum distance between the solute and the box equal to 5 Å. Subsequently, the system was neutralized with Na^+^ counterions to reproduce the physiological conditions and keep the solute molecules within the simulation box. Overall, the whole system contained 291,031 atoms. The energy minimization was carried out in two steps: (i) first with 1500 steepest descent cycles and 1000 conjugate gradient cycles with the 50 kcal/mol·Å^–2^ weight for the positional restraints on the solute, without H atoms, which were allowed to relax, and (ii) second with 6000 steepest decent cycles and 3000 conjugate gradient cycles without restraints. Later, the system was heated up to 298 K for 10 ps with the same restraints as in the second step of the minimization and equilibrated for 50 ps at 298 K with a constant pressure of 1 bar in an isothermal isobaric ensemble (NPT; N: number of particles, P: pressure, and T: temperature were kept constant). Finally, after heating, the molecular dynamic simulations (MD) were then run for 10 ns in an NPT ensemble with the PME (particle mesh Ewald method^38^) and SHAKE algorithm.^39^ The geometry of the solute obtained by averaging over structural ensembles from the last 1 ns of the production step of MD was taken for the molecular docking simulations. During this step, the collision frequency was set to 1 ps^–1^, whereas the cutoff for nonbonded interactions was set to 8 Å. All MD calculations were performed using the AMBER14 package.^40^

Molecular docking simulations were performed using AutoDock 4.2 Release 4.2.6.^41^ The structures of analytes and the receptor (DNA) without nonpolar hydrogen atoms were used. For said structures, the Gasteiger partial charges^42^ were calculated and then used in the docking simulations. The binding of analytes to the DNA helix was performed using the Genetic Algorithm. Because receptor binding sites were unknown, it was necessary to carry out docking simulations in two manners. First, less accurate docking was performed with a large grid box (96 × 126 × 54 Å^3^) and 1 Å grid point spacing, including the whole DNA strand to locate the most favorable binding sites. Subsequently, more accurate docking simulations with grid boxes covering only the most important binding sites with 0.303 Å grid point spacing were carried out to obtain more rigorous and exact results. The analyte–receptor interaction Gibbs free energy (Δ*G*) was evaluated according to eq 4:
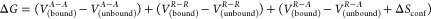
4where *A* refers to the ″analyte″ and *R* to the ″receptor″ in a docking calculation. The pairwise energy terms (*V*) include evaluations of hydrogen bonding, electrostatics, dispersion/repulsion, and desolvation. The exact form of *V* can be found in the AutoDock 4.2 manual. As can be seen from the above equation, both pairwise evaluations and the conformational entropy (Δ*S*_conf_) lost upon binding are taken into account in the assessment.

## Results and Discussion

### Optimization Based on Ethidium Bromide Interactions with DNA

**Figure 2:**
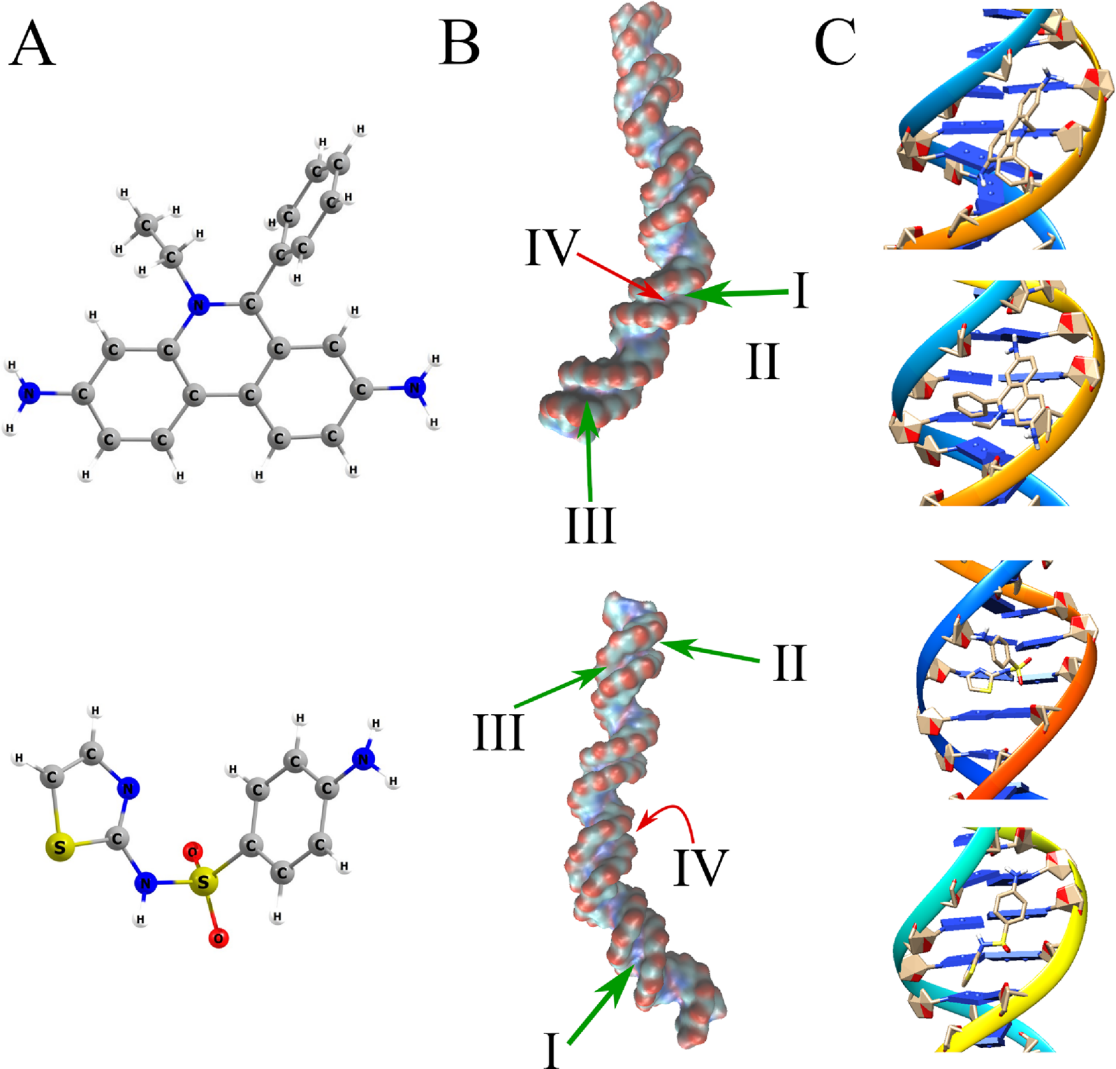
The results of theoretical calculations for ethidium (top) and sulfathiazole (bottom). (A) Equilibrium structures of *ab initio* optimized ethidium and sulfathiazole. (B) The positions of the most common binding sites (green) and the sites characterized by the highest value of analyte–receptor binding energy (red). The thickness of each arrow represents the relative abundance of a given clustering. The presented isosurface was obtained from the Gaussian density map as implemented in VMD.^50^ (C) The most popular (top) and most strongly bonded (bottom) conformation of an analyte*–*receptor complex from docking simulations focused on the most important grooves.

The results of theoretical calculations for ethidium bromide interactions with DNA are depicted on Figure 2. The equilibrium structures of *ab initio* optimized compounds and the positions of the most common (green) and most strongly bonded (red) binding sites together with the results of high-accuracy (0.303 Å grid point spacing) docking simulations are illustrated in the figure.

Ethidium bromide (EB; Figure 2A) can intercalate into a DNA duplex from the minor groove, which results in a reduction of the 36° twist to 10°, and hence, the DNA unwinds by 26°.^21,43−45^ As previously reported, the EB preferably intercalates in GC-rich sequences;^46^ however, there are also reports indicating its intercalation in AT base pairs.^47^ Intercalation has been generally considered to be the result of a hydrophobic interaction in which an aromatic molecule is drawn to a nonpolar environment of the base pairs from the hydrophilic aqueous surroundings.^48^ Moreover, computational studies with the ethidium bromide suggested that its intercalation complexes are also stabilized by frontier orbital interactions between the lowest unoccupied molecular orbital (LUMO) of the intercalator and the highest unoccupied molecular orbital (HOMO) of the adjacent purine bases.^49^ As can be seen from Figure 2B, the most preferable docking sites for ethidium are located in the bottom part of the studied DNA helix, i.e., the part closer to the anchor. Namely, both the most popular docking site (I) and the one within which analyte binds the strongest (IV) are located in the same minor groove, i.e., the second one counting from the anchored end of the double helix. The aforesaid groove is dominated by the presence of AT base pairs, which indicates a high affinity of ethidium to these two nucleobases. For the strongest bonded mode (IV), the corresponding binding energies are in the range of 4.28–5.52 kcal/mol depending on the conformation of ethidium relative to the receptor. The situation is quite different when the results of more accurate, second minor groove-focused calculations are considered. Over 90% of all dockings found exhibit binding energy over 5.5 kcal/mol. The most popular (top) and the strongest bonded (bottom) conformation of an analyte–ligand complex from docking simulations focused on the most important grooves. In the most popular bonding mode, for which the bonding energy was calculated to be equal to 6.87 kcal/mol, the ethidium is oriented perpendicularly to the minor groove. On the other hand, in the case of the complex bonded by the highest value of 7.97 kcal/mol, the molecule is oriented in a way that allows its rings to somewhat clasp one of the nucleobase pairs forming the groove, like tongs. Both the affinities of ethidium to the AT base pair and to minor groove binding overall are of no surprise as such binding modes were observed elsewhere,^45,47^ which validate the molecular docking results reported in this paper. The two remaining most popular binding sites were calculated to be placed within the first minor groove, which is not dominated by any particular base pairs as the anchored end of the helix starts with the TAG TGC sequence.

**Figure 3:**
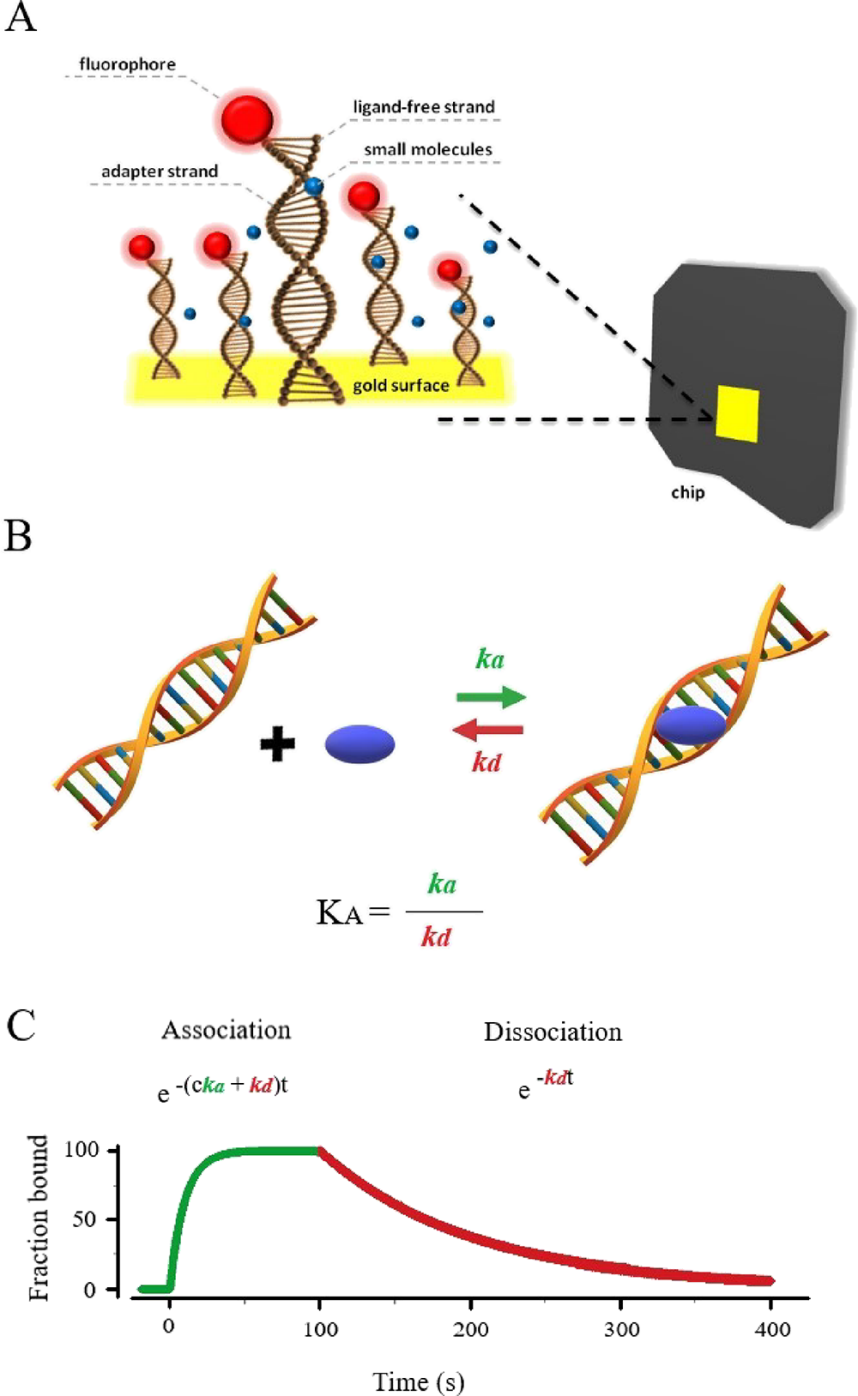
Measuring system. (A) Structure of the chip surface used for research. (B) Scheme of the occurring interaction with the assignment of *k*_a_ and *k*_d_. (C) Representative curve presenting the processes of association (green line) and dissociation (red line) registered during the measurement of the interaction kinetics.

The computational methods were used to obtain the information on which parts of the examined fragment of the DNA helix the EB molecule most preferably attaches to. The aforesaid data also indicated the binding mode, which could be further characterized by the strength of interaction and compared with the experimental value of the association constant *K*_A_. High values of *K*_A_ (binding constant, approx. 10^4^–10^6^ M^–1^) imply intercalation. The association constants for the DNA–EB adduct known in the literature differ slightly in their values from each other, depending on the measurement technique used,^19,21,51^ and are equal to about 10^5^–10^6^ M^–1^. The switchSense technique enables the description of both the association and dissociation processes occurring during the flow of the analyte by kinetic rate constants *k*_a_ (eq 1) and *k*_d_ (eq 2) and, on their basis, the determination of the association constant expressed in the form of *K*_A_ (eq 3). The curve showing the processes of association and dissociation of the analyte to the ligand system (which is a fragment of double-stranded DNA) and a diagram illustrating the ongoing process, together with the assignment of the values of *k*_a_ and *k*_d_, are presented in Figure 3 as an example.

The measurements for EB were carried out in two modes: (i) The first is dynamic, using the oscillatory movement of the helix fragments under the influence of changes in the applied voltage. The response signal is affected by a change in hydrodynamic friction as a result of analyte–ligand interaction. (ii) The second is static, in which the changes in fluorescence result only from the influence of analyte–ligand binding on the fluorophore signaling unit, causing physicochemical changes in its local environment, while DNA nanolevers are not electrically actuated in motion. Two methods (weak binders and standard kinetic, which will be discussed later in the manuscript) were used in the static mode. To determine the influence of temperature on the kinetics of the studied processes, the experiments were carried out in the three temperature variants: (i) 37 °C, which corresponds to the temperature of the human body; (ii) 25 °C, a room temperature usually kept in the chemical laboratory as well as for conducting the experiments (e.g., studies of the interaction of pharmaceuticals with DNA in solutions using instrumental techniques such as spectroscopic or electrochemical methods); and (iii) 15 °C to analyze the influence of the lowered temperature on the kinetic parameters. The duration of the association as well as dissociation processes and the flow rate values were determined based on numerous preliminary measurements (optimization of the procedure; Figure S1).

The results show that, for all measured variants, the response curves have the typical shape, and the association and dissociation processes are visible. However, at the extreme flow rate values (20, 50, and 500 μL/min), the obtained kinetic parameters were not reproducible. For this reason, the flow of 200 μL/min—neither the highest nor the lowest of the tested—has been selected for further analysis. The duration of association and dissociation processes has also been determined and is equal to 30 and 60 s, respectively. These time variants ensure complete analyte dissociation and a sufficiently long bonding time. The time of association below 10 s excluded the repeatability of the obtained results, and the time above 30 s did not affect the efficiency of the process; therefore, it was not regarded as necessary.

**Figure 4:**
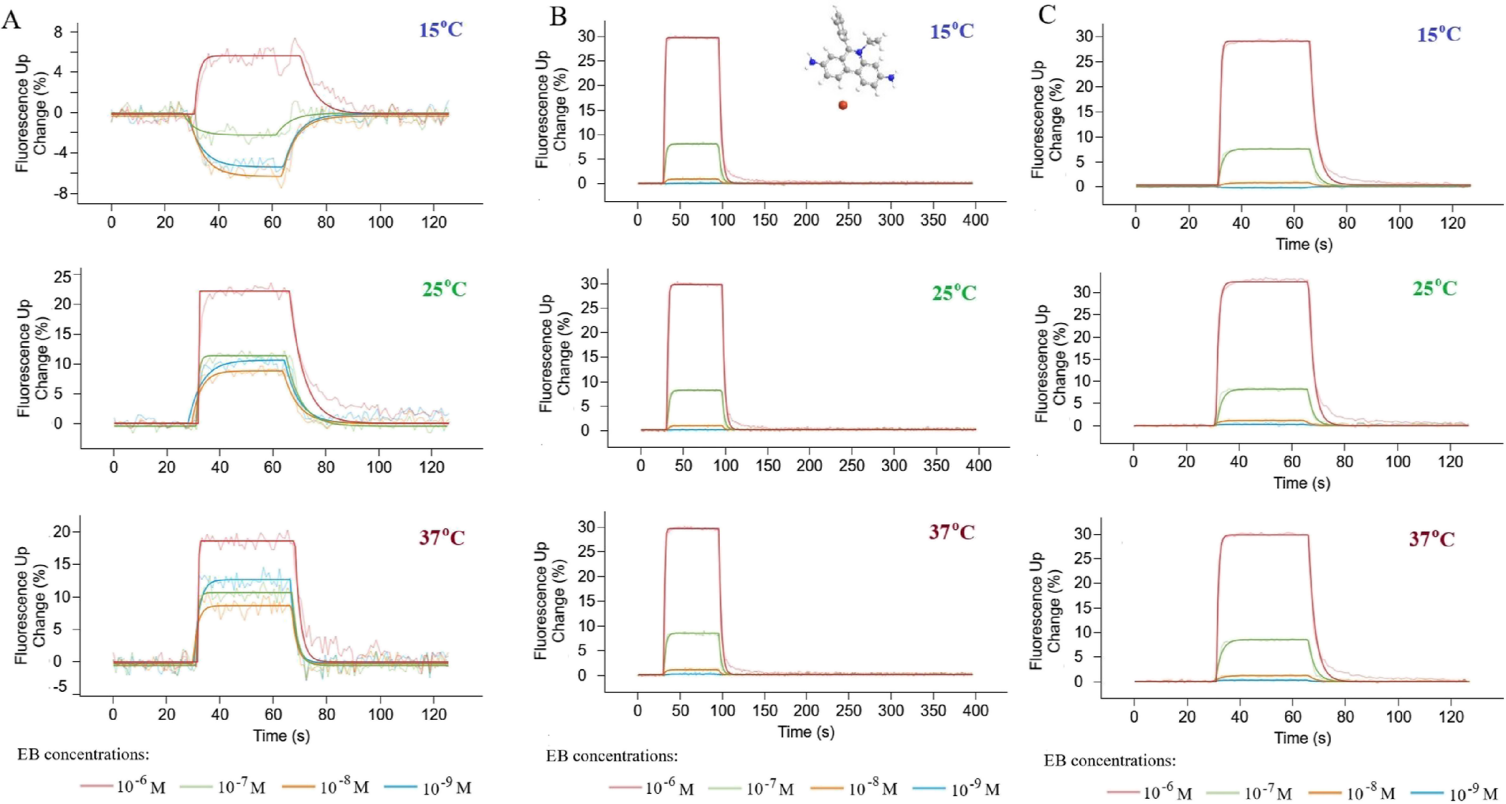
Representative results of analyses of EB interaction with DNA helix carried out in 15, 25, and 37 °C. (A) Dynamic mode, (B) static (standard) mode, and (C) static (weak binders) mode. The thinner lines in each color represent the measurement points, while the bold line represents the fitted data based on which the kinetic parameters are calculated.

Based on the optimization process, the suitability of the dynamic mode for studying the interaction of EB with the DNA chain was checked first. A detailed description of the kinetic measurement pathway in dynamic mode is described in the Experimental Section. Results of the measurements carried out in the dynamic mode at different temperatures and EB concentrations are shown in Figure 4A.

Unfortunately, the following numerous issues that disqualify the use of this mode in the studies were encountered: (i) no correlation between the concentration of the analyte and the intensity of fluorescence changes was observed, (ii) the measurements performed in this mode were not reproducible, and (iii) the values of the binding (*k*_a_) and dissociation (*k*_d_) rates obtained on their basis as well as the values of the association constant (*K*_A_) were flawed as indicated by high values of standard deviations.

Fortunately, studies on a system in which the DNA helices do not come close to the electrode surface with oscillatory movements, for which the changes in fluorescence are the result of only the binding of the analyte to DNA nanolevers, yielded much better results. In the graphs of the changes in fluorescence during the measurement (Figure 4B), a very sharp curve related to the process of association of EB to DNA was noticed. Such a shape of the slope proves that the binding is fast, even instantaneous. Carrying out the association for the next part of the minute (as assumed for this process) does not bring any significant changes in the fluorescence intensity, as evidenced by the flat segment between about 30 and 90 s of the measurement. The flow of the analyte solution through the chip was then terminated, and the flow of the buffer (without analyte) began, initiating the dissociation process. The shape of the slope representing the dissociation is less sharp, which may be due to the strong DNA–analyte interaction. For the highest applied concentration (10^–6^ M corresponding to the red line in the plots), the most elongated shape up to approx. 150 s of the measurement was observed. Ultimately, the change in fluorescence descends quite quickly to zero in all the performed measurements, which proves the complete dissociation of EB molecules from the DNA helix as a result of washing with the buffer. Therefore, chip regeneration (DNA nanolever denaturation and freeing the anchor–short single DNA strand for the next functionalization) is not required after each concentration. The determined values of *k*_a_ and *k*_d_ rates together with *K*_A_ and *K*_D_ constants were found to be repeatable (in three independent experiments) and presented in Table 1.

**Table 1:** Values (along with Their Standard Deviations in the Brackets) of Determined Association Rates (*k*_a_), Dissociation Rates (*k*_d_), Association Constants (*K*_A_), and Dissociation Constants (*K*_D_) for EB Interactions with DNA Measured by the Static Kinetic Method (switchSense Technique) in the PE140 Buffer, Flow Rate 200 μL/s

analysis mode	temp [^o^C]	*k*_a_ [M^–1^·s^–1^]	*k*_d_ [s^–1^]	*K*_D_ [M]	*K*_A_ [M^–1^]
static (standard)	15	(1.50 ± 0.16) × 10^6^	0.359 ± 0.005	(2.39 ± 0.25) × 10^–7^	(4.19 ± 0.44) × 10^6^
	25	(6.98 ± 0.63) × 10^5^	0.358 ± 0.007	(5.13 ± 0.47) × 10^–7^	(1.95 ± 0.18) × 10^6^
	37	(8.55 ± 0.95) × 10^5^	0.360 ± 0.006	(4.22 ± 0.47) × 10^–7^	(2.37 ± 0.27) × 10^6^
static (weak binders)	15	(1.69 ± 0.20) × 10^6^	0.391 ± 0.007	(2.32 ± 0.27) × 10^–7^	(4.31 ± 0.51) × 10^6^
	25	(6.04 ± 0.18) × 10^5^	0.478 ± 0.190	(7.91 ± 0.45) × 10^–7^	(1.26 ± 0.23) × 10^6^
	37	(1.03 ± 0.19) × 10^6^	0.388 ± 0.008	(3.75 ± 0.69) × 10^–7^	(2.67 ± 0.49) × 10^6^

It is apparent from Table 1 that the temperature affects the rate of the association processes taking place (see *k*_a_ values). It might be expected that the increase in the temperature would lead to higher values of the bonding rate. An increase in temperature of the environment of simple low-molecular-weight systems generally causes an increase in the mobility of mentioned systems in solutions and boosts their reactivity. The analysis of *k*_a_ values leads to the conclusion that this tendency remains in the case studied here for the temperatures within the range of 25 to 37 °C. Namely, an increase in the temperature resulted in a slight increase in the value of the association rate. On the other hand, at the temperature of 15 °C, the *k*_a_ values were comparable and even higher than for 37 °C. In the case of large biomolecular systems (such as proteins or nucleic acids), the temperature influences the conformation and thus their reactivity to a bigger extent stronger than for small molecules.^52−56^ The influence of the temperature on the conformation of biomolecules is a nontrivial problem. On this score, the increase in the temperature does not necessarily have to translate into an increase in the rate and strength of the interaction. In fact, an increase in temperature above a certain threshold may indicate that the intermolecular interactions will become even weaker. A higher temperature, leading to increased vibrations and movement of the interacting molecules, makes it difficult to maintain the interaction. It is also noteworthy that the movement of the analyte molecules during the measurement is forced by the flow rate, which affects the interaction to a bigger extent than does the temperature (at least in the studied temperature range). For that reason, it was possible to observe an acceleration of the EB to DNA association process as an effect of the temperature decrease (from 25 to 15 °C). This is presumably caused by the fact that, at 15 °C, the DNA adopts the thermodynamically favorable configuration that allows it to have a faster and stronger interaction with EB. Interestingly, the temperature did not have a large impact on the dissociation rate of the EB-DNA adduct (*k*_d_). The similar values of *k*_d_ are mainly determined by the buffer flow. Moreover, the differences in the association constant (*K*_A_) values for the measurements registered at three different temperatures are mainly a consequence of the differences in the values of the binding rate (*k*_a_). The determined kinetic parameters demonstrate that the influence of the temperature on the interaction processes involving biomolecules in the forced flow of the analyte is a complex issue. Determination of the exact dependencies and correlations would require extensive research.

The results of measurements registered in the standard stationary mode proved to be sufficiently precise to study the kinetics of EB interaction with the DNA helix. However, bearing in mind that the purpose of the study was to also describe the compounds interacting with the DNA chain to a lesser extent, our research was expanded to include also the weak binder mode in the static kinetic method. The plots of the fluorescence changes resulting from the measurements conducted in this mode are presented in Figure 4C. Due to the previously observed high rate values of association and dissociation, the duration of these consecutive processes was shortened. As expected for an EB molecule, the course of changes in fluorescence and thus the shape of the curve remained the same as in the measurements made in the standard static mode (Figure 4). This confirms the possibility of using both static mode subtypes to determine the kinetic parameters of ethidium bromide interaction with DNA.

**Figure 5:**
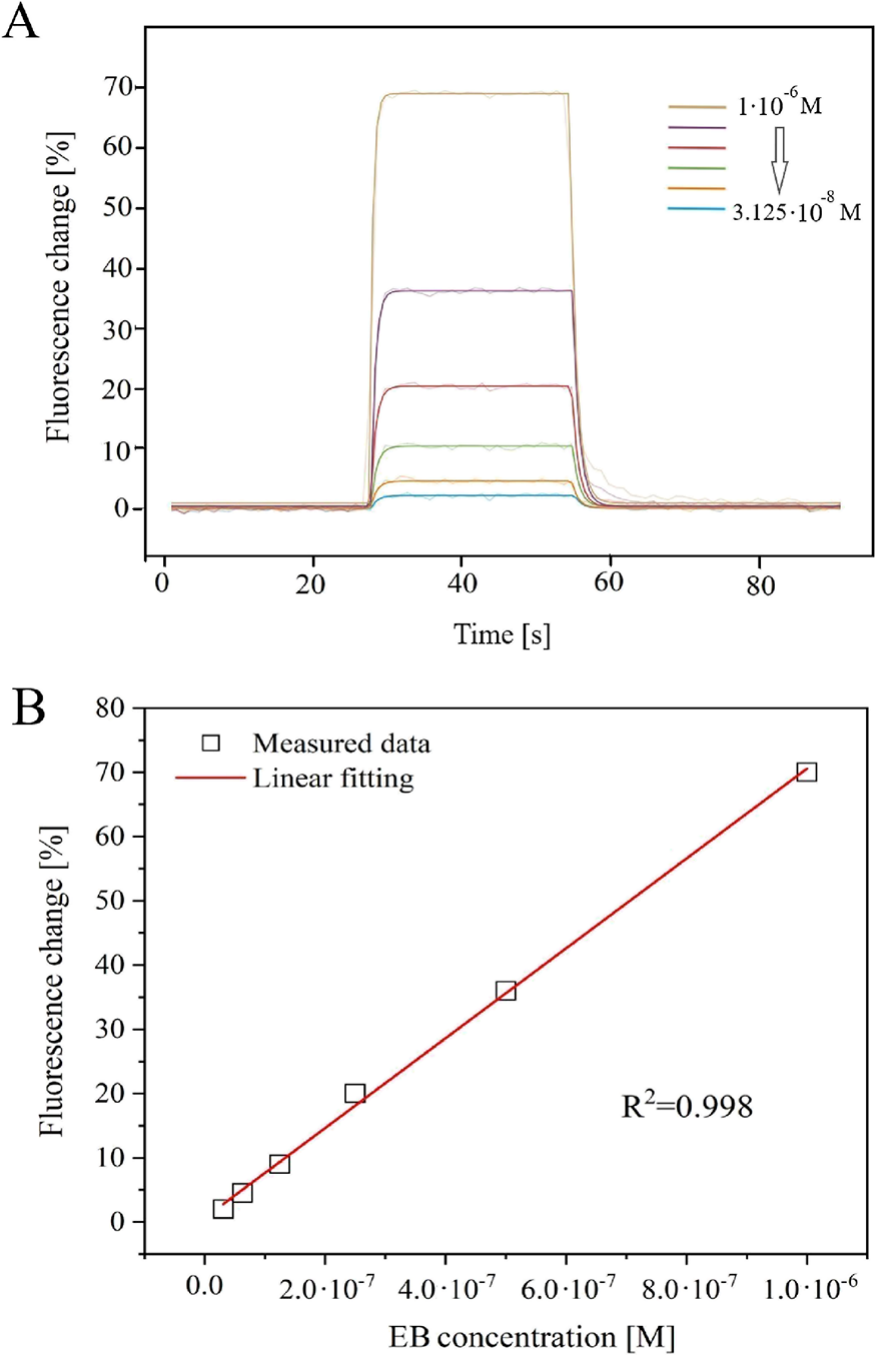
(A) Representative measurement of EB binding kinetics to DNA recorded for six concentrations (from 1 × 10^–6^ to 3.125 × 10^–8^ M, dilution factor = 2) in the PE40 buffer at 25 °C. (B) Graph of fluorescence intensity *vs* analyte concentration (EB; square points), along with linear fitting (red line; *R*^2^ = 0.998).

In the case of EB, the range of concentrations between 10^–6^ and 10^–8^ M resulted in changes in fluorescence at the level of approx. 2 to 30% in the PE140 buffer. These were the most favorable parameters for the measurements. Too low changes in fluorescence (below approx. 1%) may lead to obtaining values of kinetic parameters with a high standard deviation. The changes in the intensity of fluorescence apart from the nature of the analyte and its binding mode are also influenced by the environment in which the process takes place. Moreover, the influence of different ionic strengths on the conformation of end-tethered DNA molecules on gold surfaces has been proven.^52^ An increase in fluorophore activity was observed when registering the association of EB in the PE40 buffer (Figure 5A). The PE40 buffer is characterized by a lower NaCl concentration (lower ionic strength) compared to the PE140 one (see the Experimental Section). The change of the buffer did not significantly affect the values of the association constants (*K*_A_ = (1.52 ± 0.15) × 10^6^ M at 25 °C). Increasing the intensity of the fluorophore response through the appropriate selection of the buffer can be beneficial in the case of compounds whose binding to DNA causes only slight changes in the fluorescence of the signaling unit.

Furthermore, a linear correlation was observed between the concentration of the EB and the change in fluorescence associated with the interaction with the DNA chain (Figure 5B). This confirms the selection of appropriate parameters and the correct carrying out of the experiments.

To sum up, the results of our study for EB indicate that the dynamic method is not appropriate (not sensitive enough) for the investigation of the interactions of small molecule compounds with the nucleic acid helix. Despite our exhaustive attempts to adjust the measurement parameters, the obtained results remained unsatisfactory. The suitability of the static kinetic mode (for both standard and weak binder subtypes) for the study of the direct interaction of the EB molecule with DNA is justified and demonstrated by (i) the reproducibility of the obtained results (both the nature of the change over time and the percentage of change in fluorescence), (ii) direct correlations between the concentration of the analyte and fluorescence changes (in %), and (iii) the values of the determined *k*_a_ and *k*_d_ rates and *K*_A_ and *K*_D_ constants being reproducible with their low standard deviations.

### Sulfathiazole Binding Studies

The second small molecule considered in this study was sulfathiazole (STZ). Unlike ethidium bromide, its interaction with DNA is not widely discussed in the available literature. It was reported previously that this sulfonamide derivative interacts with DNA via binding through a helix groove.^22^ As for the docking of sulfathiazole, the situation is quite different than with ethidium bromide. This is mainly due to the significant structural differences between STZ and EB (see Figure 2A). In its equilibrium geometry, ethidium is mostly planar, whereas sulfathiazole exhibits a v-like shape (as do all remaining sulfonamides). Namely, for sulfathiazole, the most preferable docking site (I) is located in the first minor groove in the projection presented on the bottom part of Figure 2B, which is between the two minor grooves described in the case of ethidium binding (on the back of the first projection of the DNA helix). As mentioned before, this region of the double strand does not exhibit any specificity regarding the abundance of either AT or CG base pairs. The other two most favored binding modes for sulfathiazole are located on the other side of the DNA strand, i.e., in the fourth minor groove. It consists mostly of AT pairs in this case, demonstrating that the preferences of sulfathiazole toward certain nucleobases might be regarded as somewhat similar to those of ethidium. It appears from the minor-groove focused calculations that in the most popular mode of binding (6.18 kcal/mol), the v-shaped sulfathiazole molecule (the sulfonamide group CSNC dihedral angle equal to −47.43°) is parallel to the ″base″ of the minor groove. In the case of the complex bonded by the highest amount of energy (7.39 kcal/mol), the CSNC dihedral angle in sulfathiazole is equal to 164.91°, which allows for a higher contact area between two interacting systems than was the case for the most popular binding mode.

The strongest binding of sulfathiazole to the studied DNA helix, however, was found to occur within the second minor groove (bottom of Figure 2B). The energy associated with this binding was found to be somewhere in the range between 3.5 and 5.3 kcal/mol, depending on the conformation of sulfathiazole. The receptor in the region of the second minor groove is built more or less equally by both AT and CG pairs. Hence, it is rather the sequence (TCG) that this groove consists of and its closest environment that make the significant binding rather than any specific pair of nucleobases. Altogether, taking all docked conformations of both ethidium bromide and sulfathiazole into account, the average binding energy for EB was found to be ca. 0.58 kcal/mol higher than that of sulfathiazole, i.e., 7.97 *vs* 7.39 kcal/mol for ethidium and sulfathiazole, respectively. The magnitude of the difference in the average binding energy indicates a comparable affinity of the studied DNA helix toward both compounds.

The experimental results show that the fluorescence changes observed for STZ were significantly lower than those for EB, which might be due to the weaker interaction of this species with DNA, interaction in a different groove of the helix, or another bonding mode. The curves exhibited an analogous shape as in the case of EB with very low values of fluorescence changes. This suggested that the interaction constants were not possible to determine with a reasonably low standard deviation. Unfortunately, these facts expose some limitations of this method. For compounds that interact weakly with DNA (or if changes in fluorescence are very low), one can perform a qualitative analysis, i.e., answer the question of whether under given conditions the compound interacts with DNA. However, in the case of determination of the parameters (*k*_a_, *k*_d_, *K*_A_, and *K*_D_) of this interaction, it should be approached with limited confidence. A detailed discussion of the experimental results can be also found in the SI (see pages S6–S9).

### NethylS and NpropylS Binding Studies

The last research objects included in the present research were two sulfonamide derivatives differing in the length of alkylamino substituent (NethylS and NpropylS). It was demonstrated in the past that even such a small difference in their structure as a presence or absence of a −CH_2_– unit in the substituent affects acid–base and complex forming properties toward trivalent rhodium and ruthenium ions.^23^ Therefore, it seemed necessary to investigate the interactions of these compounds with the DNA chain and to check whether, in this case, we are also able to notice differences in behavior between NethylS and NpropylS. Unfortunately, determination of the binding constant of these compounds utilizing commonly used techniques such as UV–vis spectroscopy or voltamperometry turned out to be very problematic. The use of the spectrophotometric approach was prevented by the spectroscopic properties of the studied systems. The absorption maxima for NethylS, NpropylS, and DNA in the Tris buffer were located at basically the same wavelength (λ_NethylS_ = 262 nm, λ_NpropylS_ = 262 nm, and λ_DNA_ = 260 nm; Figure S3A). Spectral changes during the titration with increasing DNA concentration were limited to the increase in the intensity of the absorption maximum at λ ≅ 260 nm (Figure S3B). This was due to the overlapping of the ligand and analyte bands that made determining a reliable value of the binding constant impossible. On the other hand, the electrochemical determination was excluded from the analysis due to a very low intensity of the current peaks of studied compounds and only slight changes in these signals during the addition of the DNA solution (Figure S4).

**Figure 6:**
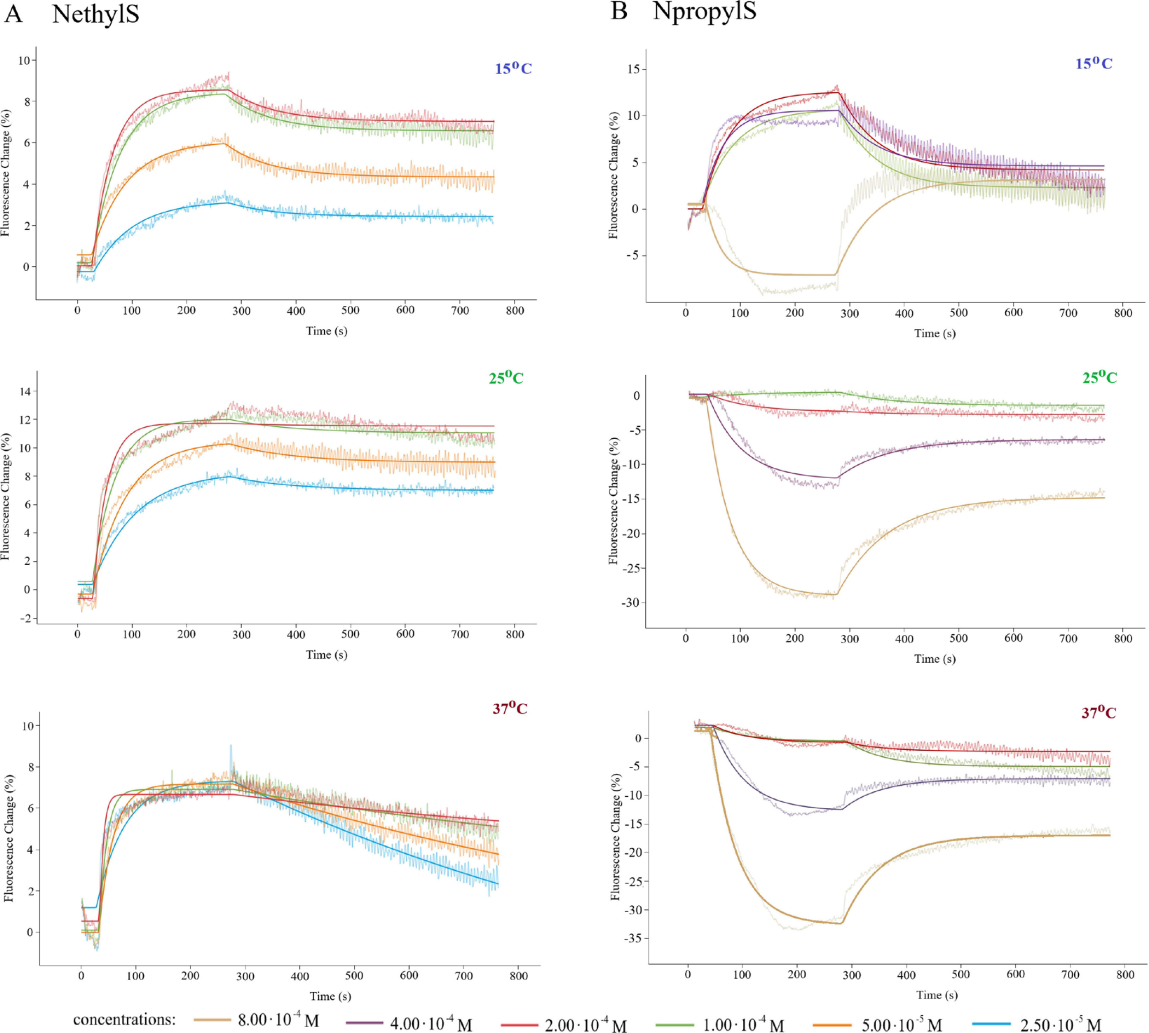
Representative results of analyses of (A) NethylS and (B) NpropylS interaction with DNA helix carried out at 15, 25, and 37 °C in static (weak binders) mode. The thinner lines in each color represent the measurement points, while the bold line represents the fitted data based on which the kinetic parameters are calculated.

To analyze the interactions of NethylS and NpropylS with DNA using the switchSense technique, we used the static (weak binder subtype) kinetic mode. The measurement parameters optimized for the first studied sulfonamide (STZ) turned out to be completely unsuitable for these two derivatives. Therefore, we were forced to determine new parameters for each studied system. In the case of the research on NethylS and NpropylS, we have noticed two essential differences in the course of the binding process of these compounds to DNA (Figure 6): (i) The association process took much longer, and the curve had a smaller slope than in the case of EB and STZ. (ii) We did not observe complete dissociation of the adduct by the buffer flow despite extending the dissociation time up to 8 min (480 s). To determine the parameters of the binding kinetics with the greatest accuracy possible, the association process was carried out for 4 min (240 s), while the data fitting was performed using a calculation model taking into account the incomplete dissociation of the analyte. This allowed us to adjust the measurement points to the calculation model. This resulted in small errors in the determined rate and constant values for the interaction of NethylS and NpropylS with the DNA chain (Table 2).

**Table 2:** Values (along with Their Standard Deviations in the Brackets) of Determined Association Rates (*k*_a_), Dissociation Rates (*k*_d_), Association Constants (*K*_A_), and Dissociation Constants (*K*_D_) for NethylS and NpropylS Interactions with DNA Measured by the Static Kinetic Method (Weak Binders) in the PE40 Buffer, Flow Rate 200 μL/S

compound	temp [^o^C]	*k*_a_ [M^–1^·s^–1^]	*k*_d_ [s^–1^]	*K*_D_ [M]	*K*_A_ [M^–1^]
NethylS	15	72.7 ± 3.6	(1.23 ± 0.04) × 10^–2^	(1.68 ± 0.10) × 10^–4^	(5.94 ± 0.35) × 10^3^
	25	77.7 ± 3.7	(8.77 ± 0.59) × 10^–3^	(1.13 ± 0.29) × 10^–4^	(8.82 ± 0.72) × 10^3^
	37	61.9 ± 2.7	(1.57 ± 0.04) × 10^–3^	(2.54 ± 0.13) × 10^–3^	(3.93 ± 0.20) × 10^3^
NpropylS	15	26.7 ± 3.4	(1.48 ± 0.05) × 10^–2^	(5.53 ± 0.73) × 10^–4^	(1.81 ± 0.24) × 10^3^
	25	16.9 ± 0.5	(1.02 ± 0.02) × 10^–2^	(6.02 ± 0.24) × 10^–4^	(1.66 ± 0.07) × 10^3^
	37	15.4 ± 0.6	(1.19 ± 0.02) × 10^–2^	(7.73 ± 0.34) × 10^–4^	(1.29 ± 0.06) × 10^3^

As for the differences between NethylS and NpropylS in the course of the interaction, the most important seems to be the decrease in signaling fluorescence (signal weakening) for the NpropylS analyte as opposed to the increase in intensity observed for NethylS. Moreover, in the case of NethylS, analyses carried out at 37 °C had led to problems with repeatability of measurements, and deviations in concentration–signal intensity dependence were observed. For both derivatives, the measurements carried out at 25 °C have shown high repeatability and reliability.

The analysis of the values of the binding rate (*k*_a_), in the case of both compounds, leads to the conclusion that the binding of the presented sulfonamides to the helix occurs much slower (about 1000× than EB and almost 100× than STZ). Therefore, these systems need much more time to interact effectively. The adduct dissociation process, described by the *k*_d_ value, also takes place much slower (10–100× compared to EB and STZ). It came as a surprise that the dissociation (especially for the NethylS–DNA adduct) was not complete even during a long process using a high buffer flow. The determined stability constant values (*K*_A_ defined as ) are in the range of 10^3^–10^4^ M^–1^, so they can be regarded as somewhat low. On the other hand, however, high resistance of the created adduct to dissociation caused by the buffer flow can be observed. This suggests a completely different mode of NethylS and NpropylS interaction with the DNA chain than in the case of EB that tends to bind quickly and strongly, but the dissociation of the adduct by the buffer flow is fast and complete. Moreover, although NethylS and NpropylS are sulfonamides, as is sulfathiazole (STZ), the nature of their interaction also seems to be significantly different. The presence of an alkylamino substituent instead of a thiazole ring in the tested analyte promotes the formation of a stable and durable adduct with DNA.

**Figure 7:**
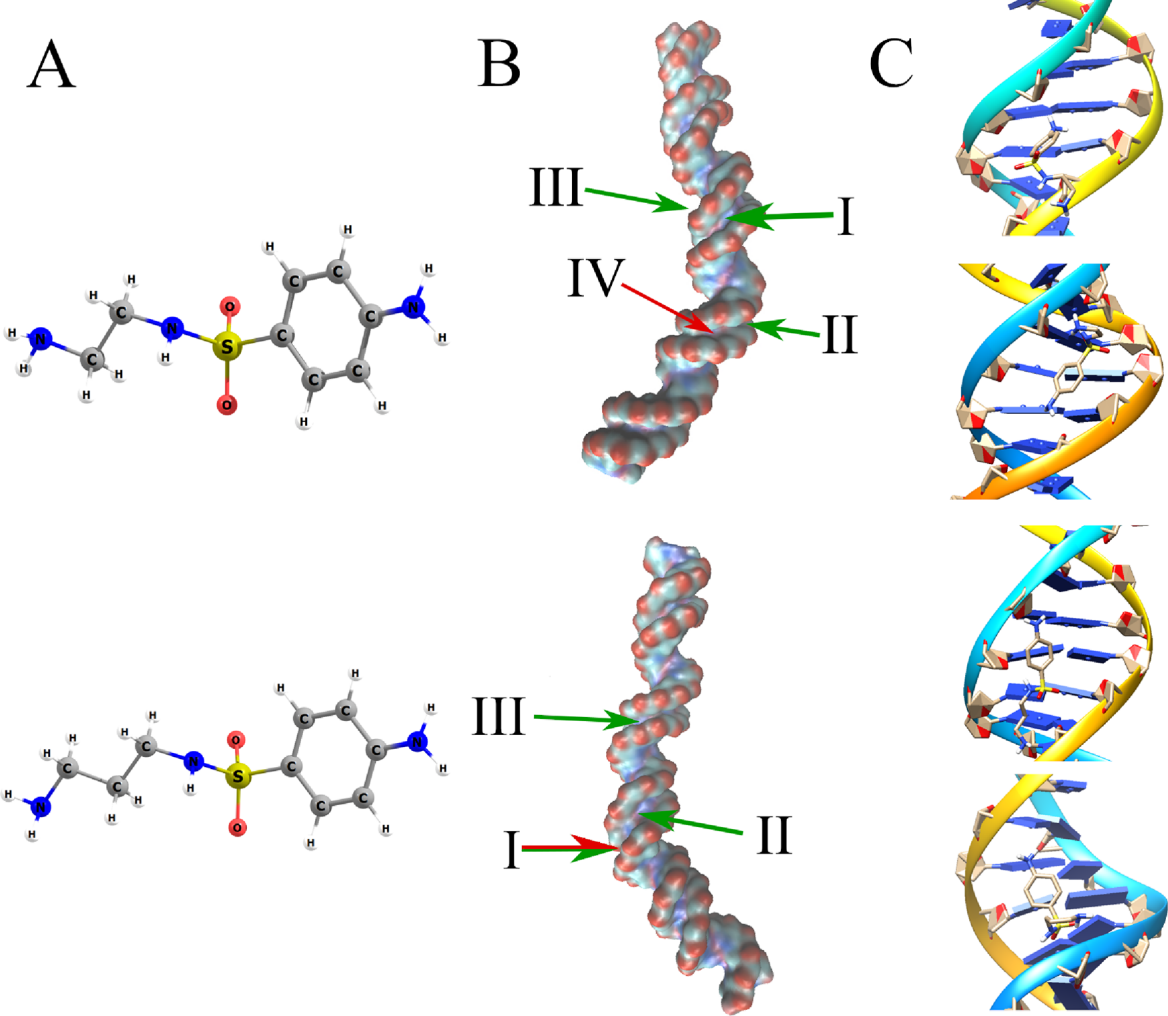
The results of theoretical calculations for NethylS (top) and NpropylS (bottom). (A) Equilibrium structures of *ab initio* optimized NethylS and NpropylS. (B) The positions of the most common binding sites (green) and the sites characterized by the highest value of analyte*–*receptor binding energy (red). The thickness of each arrow represents the relative abundance of a given clustering. The presented isosurface was obtained from the Gaussian density map as implemented in VMD.^50^ (C) The most popular (top) and most strongly bonded (bottom) conformation of an analyte*–*ligand complex from docking simulations focused on the most important grooves. For the NpropylS, both the most popular and most strongly bonded binding modes are represented by single analyte*–*receptor complex conformation (the top one). Hence, the second conformation (on the bottom) presented for NpropylS is that of the second most popular binding site.

Theoretical calculations helped explain this phenomenon. The docking simulations have revealed that for the NethylS, the two most important binding sites are located in two separate grooves, namely, the second and third ones counting from the anchor (see Figure 7). The aforesaid docking sites correspond to the most strongest and most preferred binding site, respectively. Hence, the most strongly bonded interaction of the NethylS–receptor occurs via the same groove as was the case for both EB and STZ. In said docking, the value of the CSNC angle in the NethylS analyte is equal to 123.1°, whereas that of the NCCN from the *N*-alkyl part of the analyte is equal to −4.1°, allowing for the formation of an intramolecular hydrogen bond between the H atom of the −NH_2_ group and N atom of the sulfonamide group. As for the orientation of the analyte to the receptor, the first one seems to be oriented to the two DNA strands in a parallel manner (see Figure 7). The value of the binding energy corresponding to the described docking is equal to 8.47 kcal/mol, which is higher than the corresponding value for both EB (7.97 kcal/mol) and STZ (7.39 kcal/mol). As mentioned earlier, the most popular docking site for NethylS is located in the third minor groove. In this case, the value of the CSNC dihedral angle is equal to 80.3°. Instead of the formation of intramolecular hydrogen bonds as was the case for the most strongly bonded configuration, the two H atoms of the alkyl −NH_2_ group are now involved in the formation of intermolecular hydrogen bonds with the oxygen atoms of one of the phosphate group of the receptor. These interactions are expected to stabilize the discussed configuration. The corresponding binding energy (7.40 kcal/mol) is significantly higher than both that of EB (6.87 kcal/mol) and that of STZ (6.18 kcal/mol).

The NethylS docking simulations reveal that it is expected to interact with DNA more strongly than both EB and STZ. As mentioned earlier, this is likely due to the presence of the additional −NH_2_ group in NethylS, which paves the way for the formation of three hydrogen bonds with the receptor. Contrary to the EB, the −NH_2_ group of NethylS (and NpropylS for that matter) has a significantly higher range of motion, as it is attached to the somewhat motile alkyl group. This, in turn, allows for a better adjustment of the formed hydrogen bonding net than is the case with the −NH_2_ groups of EB that are attached to the rather rigid moiety of conjugated aromatic rings.

The situation is quite similar in the case of NpropylS. Its most important docking site is located in the second minor groove, as the most preferred docking, which also happens to be the most strongly bonded one, is located there. Moreover, the second most preferred clustering also happens in the discussed groove. On the other hand, the third most favored docking site for NpropylS is located in the third minor groove. It appears from the molecular docking simulations that both NethylS and NpropylS have a high affinity toward the same parts of the studied DNA helix (second and third minor groove). This is of no surprise, as the compounds in question are structurally alike.

As it turned out from the calculations, for the NpropylS, the most popular docking and the most strongly bonded one correspond to the same configuration (see Figure 7). Hence, unlike for the remaining cases, for NpropylS, the first presented clustering corresponds to the most preferable and most strongly bonded configuration, whereas the second one corresponds to the second most preferred docking site. The DNA–NpropylS binding energy calculated for the most important (most preferred and most strongly bonded) docking was found to be equal to 8.55 kcal/mol, making the NpropylS the most strongly bonded analyte of all four studied in this paper. Not only that, but as mentioned before, the most strongly bonded configuration is also the most preferred one. In the case of the three remaining compounds, the dockings corresponding to the most strongly bonded configuration constitute only a small fraction of all clusterings. In the most important clustering, the CSNC dihedral angle in NpropylS is equal to −134.8°, and all its H atoms that are attached to the N-type hydrogen bond donors form bonds with the receptor through the O atoms of either the phosphate group or deoxyribose moiety.

The value of binding energy corresponding to the second most preferred docking site for NpropylS was calculated to be equal to 5.42 kcal/mol, which is significantly lower than the previously discussed one, although they are located in the same minor groove. The −28.5° CSNC angle in these conformations results in an L-like shape of the whole sulfonamide and enforces less favorable interaction with the receptor than was the case for the first NpropylS docking considered. As an effect of that, only some of the H atoms of the analyte are involved in hydrogen bonding with the receptor.

## Conclusions

The current paper presents another possible application and optimization of the switchSense technique for the study of the interactions of small molecules with DNA helix. This technique dominates over the other commonly used methods for the study of such interactions because it enables real-time measurements. Moreover, the immobilization of DNA to the chip surface only *via* an anchor strand (see Figure 1A) makes the DNA chain flexible. Our research confirms that the use of the measuring system shown in Figure 3A for the study of the interaction of DNA with small molecules is justified and, more importantly, brings the desired results.

The presented results confirm that the switchSense technique can be used to study DNA–small molecule interactions only in the static mode. In the experiments performed with EB, the changes in fluorescence were high, confirming the EB–DNA adduct formation as well as the dissociation process. The results show a linear dependence between the analyte concentration and signal changes. The experiments were repeated (at least three times), and dissociation and association constants with acceptable values of standard deviation were obtained. The results of molecular docking simulations performed for studied compounds provided additional information. These results indicate that both the most preferable and the one within which EB interacts with the strongest docking site are in the same minor groove (the second one from the anchor part). EB showed a significant affinity to the AT pairs, which are abundant in the aforesaid groove.

Unfortunately, the switchSense technique is limited, which has been noticed in the case of the second analyzed compound, STZ, for which interaction with DNA caused rather modest changes in the fluorophore signal and whose bonding was much weaker than EB. In this case, the conducted experiments allowed us to conclude that although STZ interacts with DNA, the procured association and dissociation constant values are affected by the substantial error. To further our research, we are planning on solving this obstacle. In the case of STZ, the most preferable docking site was found to occur in the first minor groove (from the anchor side). However, it was established that the STZ binds the strongest to the studied DNA within the second minor groove. It was also found that the average compound–DNA binding energy of EB was higher than that of STZ, which may explain the results of the conducted experiments for the DNA–STZ system.

The research results obtained for NethylS and NpropylS turned out to be both compelling and promising. It has been demonstrated that both of these compounds bind to the DNA helix rather slowly, but the adduct formed in the process is stable and resistant to dissociation forced by buffer flow. This might be due to the formation of hydrogen bonds as a result of interaction with DNA. The applied theoretical model of incomplete dissociation has allowed for both a great fit to the measuring points and obtainment of the values of kinetic parameters with small standard deviations. The collected results predispose NethylS and NpropylS as decent candidates for further biological research to determine their antimicrobial and anticancer activity.

Taken together, our findings suggest that the switchSense technique is a decent alternative to the previously used methods of studying the interactions of small compounds with DNA. The switchSense technique provides information on both binding rate (*k*) and binding constant (*K*), whereas methods such as voltammetric or spectroscopic methods provide data on the latter only. The knowledge of binding rates gives a much deeper insight into ongoing processes.

## Authors’ Contribution Statement

S. Ramotowska: conceptualization of part of kinetic measurements, determination of kinetic parameters for ethidium bromide, writing a part of the original draft. P. Spisz: conceptualization of part of kinetic measurements, determination of kinetic parameters for sulfathiazole, writing a part of the original draft. J. Brzeski: conceptualization and investigation of the computational part of studies, writing a part of the original draft. A. Ciesielska: determination of kinetic parameters, spectroscopic and voltammetric studies for NethylS and NpropylS. M. Makowski: conceptualization, supervision, funds acquisition, writing – review and editing.

## Supporting Information Available

The Cartesian coordinates (in Å) together with electronic energies (*E*), enthalpies (*H*), and Gibbs free energies (*G*) (in a.u.) corresponding to the studied ligands (Table S1). Example graphs of EB (concentration range: 1 × 10^–6^–3.125 × 10^–8^ M) kinetic measurements at various flow rate values and time variants of association (A) and dissociation (D) processes, used to select the optimal measurement conditions. (A) 20 μL/s, A: 35 s, D: 70 s; (B) 50 μL/s, A: 30 s, D: 60 s; (C) 100 μL/s, A: 25 s, D: 50 s; (D) 200 μL/s, A: 20 s, D: 40 s; (E) 400 μL/s, A: 15 s, D: 30 s; and (F) 500 μL/s, A: 10 s, D: 20 s (Figure S1). Sulfathiazole binding studies—experimental studies. Representative results of analyses of STZ interaction with DNA helix carried out at 15, 25, and 37 °C: (A) static (standard) mode and (B) static (weak binders) mode. The thinner lines in each color represent the measurement points, while the bold line represents the fitted data based on which the kinetic parameters are calculated (Figure S2). Values (along with their standard deviations in the brackets) of determined association rates (*k*_a_), dissociation rates (*k*_d_), association constants (*K*_A_), and dissociation constants (*K*_D_) for STZ interactions with DNA measured by the static kinetic method (switchSense technique) in the PE40 buffer, flow rate 200 μL/s (Table S2). Spectroscopic and voltammetric studies—experimental. (A) Spectra of DNA (red line; *c* = 2.5 × 10^–4^ M), NethylS (black line; *c* = 7.2 × 10^–5^ M), and NpropylS (blue line; *c* = 7.2 × 10^–5^ M) solutions registered in Tris buffer at pH 7.4. (B) The titration spectral curves obtained for NethylS with different DNA concentrations (from 0 to 80 μm). The arrow shows the direction of change upon the increase of DNA (Figure S3). Cyclic voltammograms registered for 2 × 10^–4^ M NethylS in the absence and the presence of 20–200 μM ct-DNA on the glassy carbon electrode. Scan rate: 100 mV/s, temperature: 25 °C (Figure S4) (PDF)
