# Supplementary material for: Application of the SwitchSense Technique for the Study of Small Molecules’ (Ethidium Bromide and Selected Sulfonamide Derivatives) Affinity to DNA in Real Time
Source: J Phys Chem B. 2022 Sep 15;126(38):7238–51. doi: 10.1021/acs.jpcb.2c03138 (PMC9527753; doi:10.1021/acs.jpcb.2c03138)
Supplement: Supplementary file 1 — jp2c03138_si_001.pdf [file jp2c03138_si_001.pdf]

Supporting Information

Application of the SwitchSense Technique for the Study of Small Molecules (Ethidium Bromide and Selected Sulfonamide Derivatives) Affinity to DNA in Real-Time

Sandra Ramotowska[1#], Paulina Spisz[1#], Jakub Brzeski[1,2], Aleksandra Ciesielska[1], Mariusz Makowski[1*]

[1]Faculty of Chemistry, University of Gdańsk, Wita Stwosza 63, 80-308 Gdańsk, Poland

[2]Department of Chemistry, University of Pittsburgh, Pittsburgh 15260 Pennsylvania, USA

[#]equal contribution

[*]Corresponding author: mariusz.makowski@ug.edu.pl

Table S1. The cartesian coordinates (in Å) together with electronic energies (E), enthalpies (H) and Gibbs free energies (G) (in au) corresponding to the studied ligands.

System	Coordinates			
**EB**<br>E = -976.3190512<br>H = -975.928345<br>G = -975.997425	N	0.509786	-1.057338	0.151739
	N	5.325094	-1.480261	0.130972
	N	-2.852014	3.969046	-0.237649
	C	-0.601045	-0.325810	0.096577
	C	1.786174	-0.476717	0.119235
	C	-0.552575	1.087927	0.014400
	C	1.889301	0.923764	0.042945
	C	0.706116	1.725278	-0.009277
	C	0.409393	-2.535321	0.220271
	C	-1.922679	-1.008775	0.122120
	C	2.934473	-1.278767	0.163762
	C	-1.748622	1.838110	-0.041452
	C	3.189592	1.474653	0.019807
	C	0.715983	3.133919	-0.089077
	C	4.196595	-0.710117	0.137042
	C	-1.713173	3.211530	-0.123307
	C	0.592108	-3.189845	-1.139467
	C	4.305040	0.697455	0.064077
	C	-0.444717	3.846012	-0.146011
	C	-2.529850	-1.392044	-1.068652
	C	-2.572358	-1.214641	1.335748
	C	-3.775692	-2.001399	-1.042071
	C	-3.816239	-1.826150	1.355747
	C	-4.416616	-2.222407	0.168389
	H	1.141932	-2.876529	0.945052
	H	-0.565162	-2.776544	0.625288
	H	2.866722	-2.352779	0.200779
	H	-2.699183	1.326071	-0.026615
	H	3.313145	2.544989	-0.035148
	H	1.649535	3.674108	-0.109575
	H	-0.219425	-2.911776	-1.809582
	H	0.574094	-4.271938	-1.019644
	H	1.534730	-2.911370	-1.606821
	H	5.284657	1.155395	0.038708
	H	-0.401067	4.925559	-0.215298
	H	-2.037062	-1.202901	-2.013231
	H	-2.105929	-0.897547	2.259359
	H	-4.246595	-2.298630	-1.968462
	H	-4.317821	-1.989739	2.299154
	H	6.205391	-1.041797	0.330593
	H	5.259484	-2.444653	0.400242

	H	-3.726721	3.542471	0.009932
	H	-2.790874	4.945379	-0.011596
	H	-5.387159	-2.697833	0.186603
**STZ**<br>E = -1459.4137777<br>H = -1459.216595<br>G = -1459.275309	S	-0.082353	1.805926	0.301655
	S	-3.288384	-0.750839	-1.073233
	O	0.157887	2.941932	-0.546481
	O	-0.379724	1.943261	1.691701
	N	-1.415068	1.102374	-0.447914
	N	4.328323	-2.076452	-0.374443
	N	-1.617793	-0.843021	0.883296
	C	1.233445	0.659496	0.110585
	C	2.004928	0.688693	-1.044432
	C	1.498515	-0.263794	1.113210
	C	3.321098	-1.147355	-0.199511
	C	3.040203	-0.208677	-1.199628
	C	2.535688	-1.159590	0.958164
	C	-1.976160	-0.111612	-0.116449
	C	-3.329248	-2.119173	-0.014245
	C	-2.388017	-1.979576	0.942397
	H	1.804173	1.427155	-1.807613
	H	0.886304	-0.274370	2.001942
	H	-1.576595	1.477431	-1.367116
	H	3.644196	-0.184284	-2.097191
	H	2.742774	-1.881021	1.737730
	H	5.042401	-1.841687	-1.041216
	H	4.690208	-2.504989	0.459260
	H	-4.031775	-2.919720	-0.161278
	H	-2.206141	-2.692236	1.731850
**NethylS**<br>E = -1025.5612008<br>H = -1025.324973<br>G = -1025.382999	S	-0.716619	0.437489	0.468933
	O	-0.997711	1.837613	0.223915
	O	-1.093646	-0.108506	1.755207
	N	5.044229	-0.473514	-0.509299
	C	0.992525	0.172542	0.170306
	C	1.550794	-1.075832	0.434273
	C	1.786951	1.208690	-0.301178
	C	3.713726	-0.247809	-0.252427
	C	2.895087	-1.285248	0.225597
	C	3.136136	1.002048	-0.511355
	H	0.931667	-1.881621	0.803287
	H	1.348306	2.175586	-0.499496
	H	3.327387	-2.255387	0.430522
	H	3.752780	1.811353	-0.878439
	H	5.471479	-1.256542	-0.045098
	H	5.640615	0.334213	-0.564481
	N	-1.424395	-0.544794	-0.656219
	H	-1.277695	-0.162272	-1.582442
	C	-2.801347	-0.988295	-0.421774

	H	-2.841451	-1.466582	0.554189
	H	-3.000418	-1.756896	-1.169596
	C	-3.855033	0.109945	-0.514714
	H	-3.702290	0.826377	0.293115
	H	-3.721487	0.653622	-1.457952
	N	-5.183303	-0.477884	-0.364699
	H	-5.422593	-1.009858	-1.192295
	H	-5.878302	0.252412	-0.288070
**NpropylS**<br>E = -1064.8759518<br>H = -1064.609559<br>G = -1064.671105	S	-0.291070	0.373152	0.508222
	O	-0.594150	1.783396	0.371544
	O	-0.645250	-0.275529	1.752789
	N	5.477337	-0.367745	-0.572139
	C	1.420633	0.160079	0.179950
	C	2.005812	-1.089263	0.370103
	C	2.189707	1.235395	-0.243057
	C	4.144887	-0.182783	-0.291581
	C	3.351722	-1.260444	0.137552
	C	3.540304	1.067032	-0.477852
	H	1.406360	-1.925738	0.701480
	H	1.730730	2.202880	-0.384270
	H	3.805124	-2.231273	0.286078
	H	4.137051	1.906974	-0.807053
	H	5.923783	-1.164731	-0.151724
	H	6.056963	0.453867	-0.587230
	N	-0.994047	-0.531583	-0.682453
	H	-0.886319	-0.060903	-1.572706
	C	-2.352431	-1.041029	-0.459292
	H	-2.338139	-1.609574	0.468873
	H	-2.536566	-1.750078	-1.266735
	C	-3.448840	0.016754	-0.424253
	H	-3.272032	0.709924	0.400018
	H	-3.415344	0.601415	-1.348285
	C	-4.826609	-0.609299	-0.268149
	H	-4.983899	-1.338346	-1.073032
	H	-4.867084	-1.162978	0.672253
	N	-5.863513	0.423265	-0.229000
	H	-6.770110	-0.005454	-0.094748
	H	-5.907755	0.894446	-1.124509

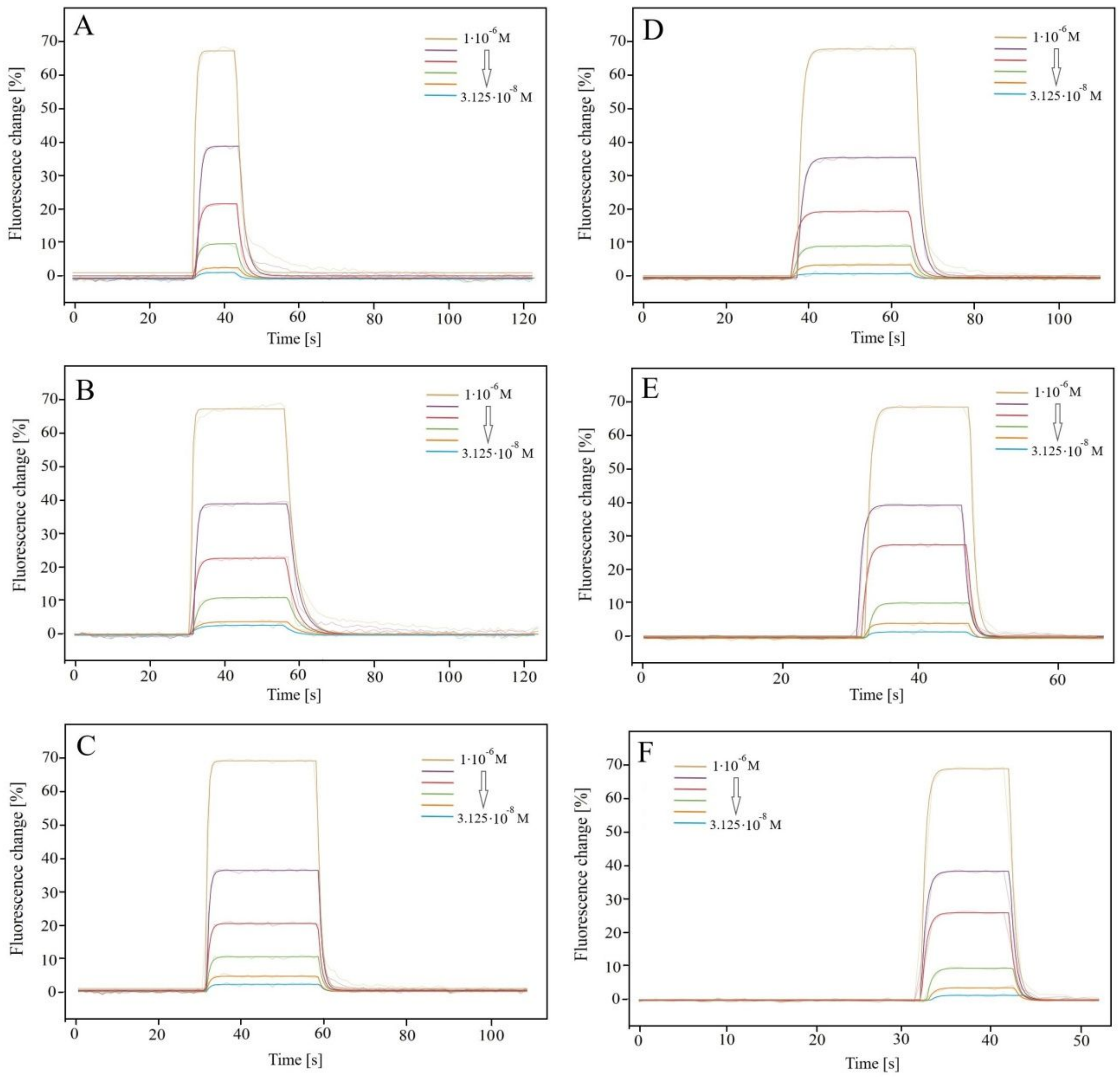

Figure S1. Example graphs of EB (concentration range: $1\cdot10^{-6}$–$3.125\cdot10^{-8}$ M) kinetics measurements at various flow rate values and time variants of association (A) and dissociation (D) processes, used to select the optimal measurement conditions. **A.** 20 μL/s, A: 35 s, D: 70 s, **B.** 50 μL/s, A: 30 s, D: 60 s, **C.** 100 μL/s, A: 25 s, D: 50 s, **D.** 200 μL/s, A: 20 s, D: 40 s, **E**. 400 μL/s, A: 15 s, D: 30 s, **F**. 500 μL/s, A: 10 s, D: 20 s.

1. **Sulfathiazole binding studies – experimental studies**

After analyzing the results for EB and STZ in both static and dynamic modes, it has been decided to carry out further research in the static mode only, using the same two methods: (i) standard kinetic, in which the association and dissociation times were 60 s and 300 s, respectively, and (ii) the weak binders method, dedicated for compounds interacting with DNA to a lesser extent, for which mentioned values were equal to 30 s and 60 s, respectively. The samples were prepared and analyzed at five concentration variants 0 (for the control), $1 \cdot 10^{-4}$, $5 \cdot 10^{-5}$, $2.5 \cdot 10^{-5}$, $1.25 \cdot 10^{-5}$ M. The concentration selection resulted from the differences in response (fluorescence change), as a consequence of weaker interaction of STZ–DNA than in the case of ethidium bromide. It was also not possible to obtain higher concentration values due to the limited solubility of the STZ compound. The research carried out for EB has also shown a significant influence of the reaction environment. Better results were obtained for measurements carried out in the PE40 buffer, therefore it was also used in this case. The same measurement system (see **Figure 3A**) and temperature variants (15, 25, and 37ºC) were used during the experiment. The results were analyzed using the mathematical model (1:1).

The representative results of the measurements obtained for the STZ are presented in **Figure S2**. It should be noticed that in this case the changes in fluorescence, as compared to EB, are much smaller – equal to ca. 5% for the highest concentration (10^{-4} M of STZ). Fluorescence changes are directly transferable into the values of the obtained constants and are thousand times lower than for EB with large standard deviations (see **Table S2**).

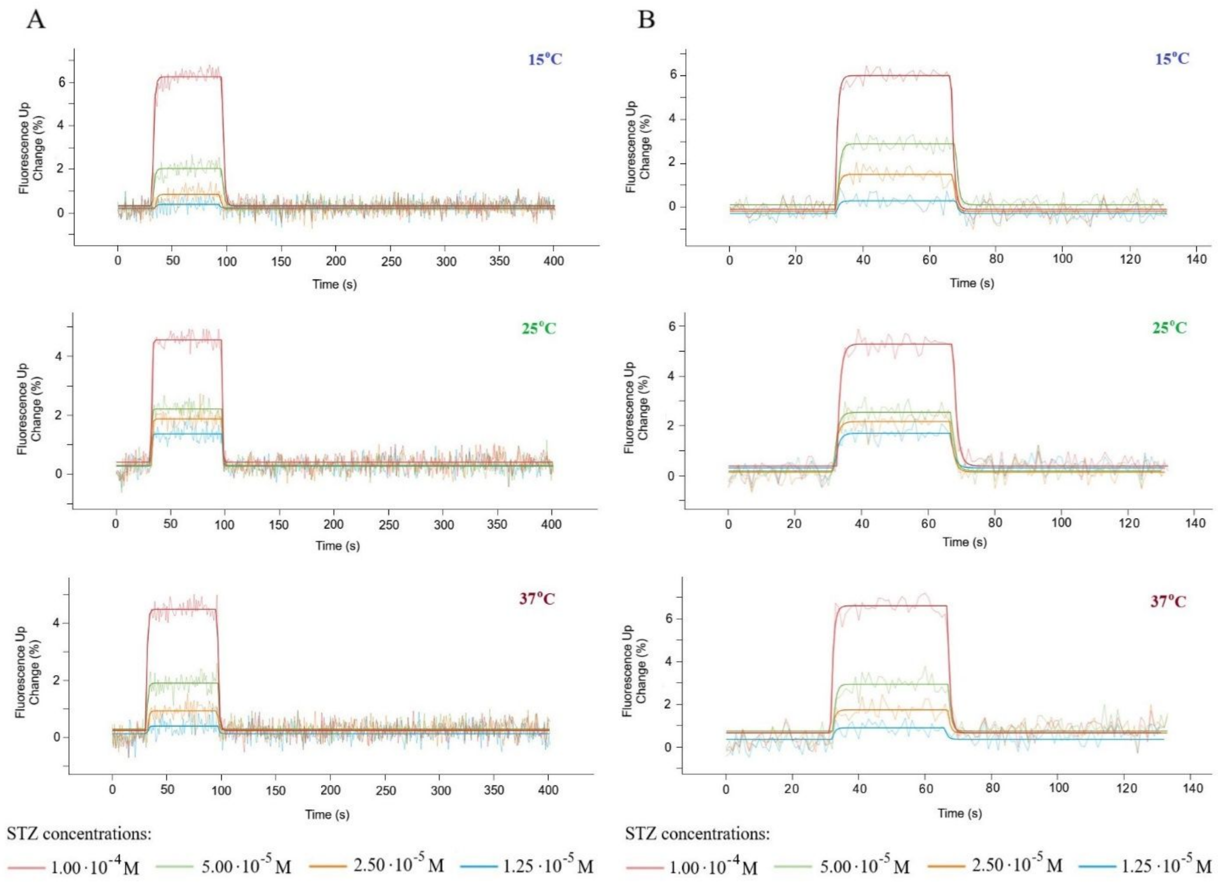

Figure S2. Representative results of analyzes of STZ interaction with DNA helix carried out at 15, 25, and 37°C. **A.** Static (standard) mode, **B.** Static (weak binders) mode. The thinner lines in each color represent the measurement points, while the bold line represents the fitted data based on which the kinetics parameters are calculated.

Table S2 Values (along with their Standard Deviations in the brackets) of Determined Association Rates (k_a), Dissociation Rates (k_d), Association Constants (K_A), And Dissociation Constants K_D For STZ Interactions with DNA Measured by Static Kinetic Method (Switchsense Technique) in PE40 Buffer, Flow Rate 200 µL/s.

**Analysis mode**	**Temp [°C]**	**k_a [$M^{-1}\cdot s^{-1}$]**	**k_d [s^{-1}]**	**K_D [M]**	**K_A [M^{-1}]**
**Static (Standard)**	**15**	$(6.11 \pm 4.29)\cdot 10^3$	$906 \pm 182\cdot 10^{-3}$	$148 \pm 108\cdot 10^{-6}$	**$(6.74 \pm 4.93)\cdot 10^3$**
	**25**	$(2.71 \pm 2.85)\cdot 10^3$	$783 \pm 87\cdot 10^{-3}$	$289 \pm 306\cdot 10^{-6}$	**$(3.46 \pm 3.66)\cdot 10^3$**
	**37**	$(0.66 \pm 0.24)\cdot 10^3$	$960 \pm 8\cdot 10^{-3}$	$146 \pm 54\cdot 10^{-6}$	**$(6.87 \pm 2.55)\cdot 10^3$**
**Static (Weak binders)**	**15**	$(1.47 \pm 1.77)\cdot 10^3$	$937 \pm 106\cdot 10^{-3}$	$636 \pm 768\cdot 10^{-6}$	**$(1.57 \pm 1.90)\cdot 10^3$**
	**25**	$(2.44 \pm 1.27)\cdot 10^3$	$770 \pm 53\cdot 10^{-3}$	$315 \pm 165\cdot 10^{-6}$	**$(3.17 \pm 1.66)\cdot 10^3$**
	**37**	$(1.61 \pm 1.83)\cdot 10^3$	$376 \pm 69\cdot 10^{-3}$	$234 \pm 271\cdot 10^{-6}$	**$(4.27 \pm 4.94)\cdot 10^3$**

Despite much lower response values, the curves retained their typical shape, and the consecutive processes of STZ–DNA complex formation and its complete dissociation are visible (see **Figures 3B** and **C**). Surprisingly, despite the weaker interaction of STZ with DNA, the self-processes of association and dissociation were rapid, as evidenced by the very sharp curve. The measurements with the use of two methods in the static mode confirmed the facts observed in the case of EB. There were no clear differences between the results obtained in the standard kinetics and those with the weak binders methods.

2. Spectroscopic and voltammetric studies - experimental

The *Calf thymus* DNA (*ct*-DNA) was purchased from Sigma-Aldrich. The synthesis pathway for NethylS and NpropylS procedure were described elsewhere[1]. Electronic absorption spectra were registered with an Evolution 300 (Thermo Scientific) spectrophotometer in the range of 215 – 350 nm at 25°C. Spectrophotometric titrations were carried out by gradually increasing the concentration of *ct*-DNA from 0 to 80 μM. The analyte was the 2 mL of $7.2 \cdot 10^{-5}$ M compound (NethylS/NpropylS) to which 1 mL of *ct*-DNA solution ($c = 2.5 \cdot 10^{-4}$ M) was gradually added, from the syringe (1 mL; Hamilton). The measurements were repeated two times, each consisting of 120 steps. Electrochemical measurements were carried out using an Autolab PGSTAT204 potentiostat/galvanostat (Metrohm Autolab B.V., The Netherlands), which is controlled by the Nova software. A single-compartment, three-electrode cell was used for all voltametric measurements. The working electrode was a glassy carbon (GC) electrode of 2 mm diameter. The electrode was carefully polished before each experiment using a 0.5 μm alumina suspension (Buehler). A platinum wire served as an auxiliary electrode. All potentials were measured using a stable silver/silver chloride (Ag/AgCl) electrode in an aqueous sodium chloride solution (1 M NaCl) as a reference electrode. All cyclic voltammetry (CV) measurements were performed at a temperature of 25°C, and the solutions were degassed by passing argon. At the start of the experiment, the cell was held at the start potential for 10 s of silence time. The reproducibility of the results was ensured by recording the voltammograms several times for each experiment. The series of NethylS/NpropylS solutions with increasing concentrations of DNA (from 0 to 200 μM) were prepared in Tris buffer, at a constant concentration of the compound ($2 \cdot 10^{-4}$ M).

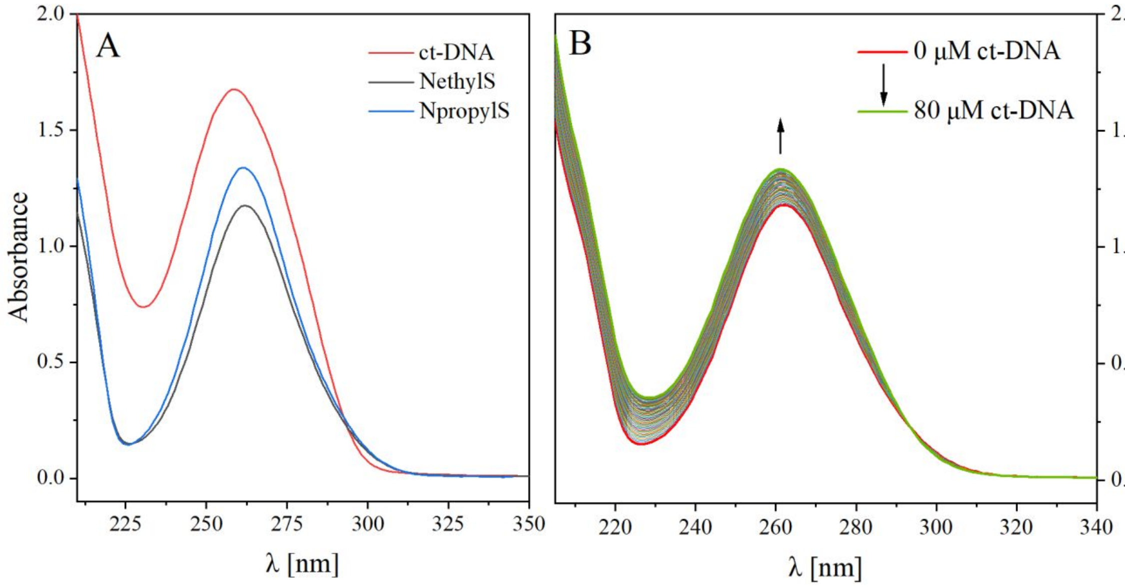

Figure S3. **A.** Spectra of: DNA (red line; c = 2.5·10^{-4} M), NethylS (black line; c = 7.2 ·10^{-5} M), NpropylS (blue line; c = 7.2 ·10^{-5} M) solutions registered in Tris buffer at pH 7.4. **B.** The titration spectral curves obtained for NethylS with different DNA concentrations (from 0 to 80 µm). Arrow shows the direction of change upon the increase of DNA.

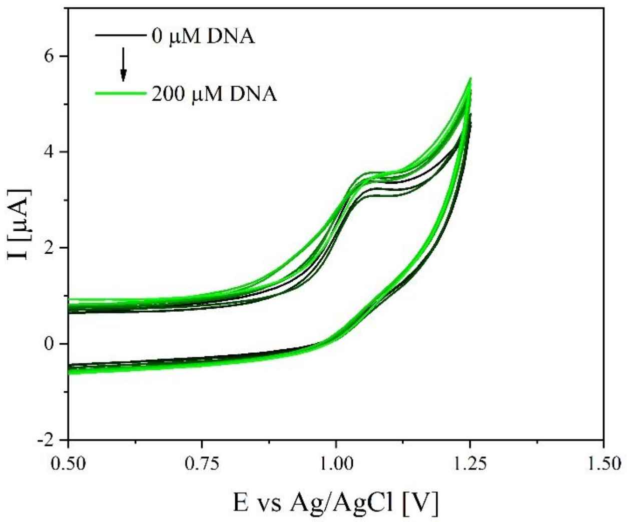

Figure S4. Cyclic voltammograms registered for 2·10^{-4} M NethylS in the absence and the presence of 20 – 200 µM *ct*-DNA on the glassy carbon electrode. Scan rate: 100 mV/s, temperature: 25 °C.

References

1. Ciesielska, A.; Gawrońska, M.; Makowski, M.; Ramotowska, S. Sulfonamides Differing in the Alkylamino Substituent Length – Synthesis, Electrochemical Characteristic, Acid-Base Profile and Complexation Properties. *Polyhedron* **2022**, *221*, 115868. https://doi.org/10.1016/j.poly.2022.115868.
